# Coleopteran Antimicrobial Peptides: Prospects for Clinical Applications

**DOI:** 10.1155/2012/101989

**Published:** 2012-03-01

**Authors:** Monde Ntwasa, Akira Goto, Shoichiro Kurata

**Affiliations:** ^1^School of Molecular and Cell Biology, University of the Witwatersrand, Wits 2050, South Africa; ^2^Graduate School of Pharmaceutical Sciences, Tohoku University, Aoba 6-3, Aramaki, Aoba-ku, Sendai 980-8578, Japan

## Abstract

Antimicrobial peptides (AMPs) are activated in response to septic injury and have important roles in vertebrate and invertebrate immune systems. AMPs act directly against pathogens and have both wound healing and antitumor activities. Although coleopterans comprise the largest and most diverse order of eukaryotes and occupy an earlier branch than *Drosophila* in the holometabolous lineage of insects, their immune system has not been studied extensively. Initial research reports, however, indicate that coleopterans possess unique immune response mechanisms, and studies of these novel mechanisms may help to further elucidate innate immunity. Recently, the complete genome sequence of *Tribolium* was published, boosting research on coleopteran immunity and leading to the identification of *Tribolium* AMPs that are shared by *Drosophila* and mammals, as well as other AMPs that are unique. AMPs have potential applicability in the development of vaccines. Here, we review coleopteran AMPs, their potential impact on clinical medicine, and the molecular basis of immune defense.

## 1. Overview

Research on innate immunity has led to an accumulation of information that offers prospects for the development of antimicrobial therapeutic drugs and vaccines. The low rate of discovery of new antibiotics, the emergence of multiple-drug resistance, and the alarming death rate due to infection indicate a clear need for the development of alternative means to combat infections. A highlight of the 20th century was the discovery of vaccines that led to the eradication of diseases such as polio, small pox, and others. Even after more than two decades, however, a vaccine against the highly mutable human immune-deficiency virus remains to be developed, illustrating the need for new strategies to produce vaccines. A better understanding of innate immunity has revealed important links between innate and adaptive immune systems that could lead to effective approaches in vaccine development.

Coleopterans comprise 40% of the 360,000 currently known insect species and are therefore the largest and most diverse order of eukaryotic organisms [1]. *Tribolium*, the coleopteran model, is proposed to be a better model than *Drosophila,* especially for evolutionary studies, as it is acknowledged to be the most evolutionarily successful metazoan and to be more representative of insects in general than *Drosophila* [1, 2]. Coleopterans, with no adaptive immunity, thrive on this planet. Studies of the molecular basis of coleopteran immunity could therefore lead to a better understanding of the evolution of the innate immune system. Much of the work on innate immunity and studies of the functional aspects of antimicrobial peptides (AMPs) has been performed using *Drosophila,* which represents dipterans, while studies on coleopterans lag behind. Insects and humans share innate immunity, but humans also have adaptive immunity. Some of the conserved molecular signaling pathways that are used by insects and humans for immune defense are also used for early embryonic development in insects, but there are notable differences, probably due to the fact that the innate immune systems of invertebrates and vertebrates diverged some 800 million years ago, and adaptive immunity appeared in the vertebrate branch only about 500 million years ago [3, 4]. The divergence of dipterans and coleoptera occurred some 284 million years ago, and *Drosophila*, in the dipteran branch, exhibits a remarkably accelerated protein evolution [5]. Furthermore, despite these separate evolutionary paths, molecular coevolution could have occurred between coleoptera and mammals due to interdependence, that is, sharing common habitats and resources.

While the majority of the work on immunity has been conducted using *Drosophila* as a model, there is evidence that coleoptera has retained many ancestral vertebrate genes, suggesting that studies of coleoptera could provide more insight into the properties and evolution of innate immunity. For example, *Tribolium* has many ancestral genes that are present in vertebrates and absent in *Drosophila* [6]. Similarly, the sequenced *Tribolium* genome revealed that ancestral genes involved in cell-cell communication and development are retained in *Tribolium,* but not in *Drosophila* [2]. Furthermore, in homology searches, human genes compare significantly better with *Tribolium* than *Drosophila* [5].

AMPs are small peptides characterized by an overall positive charge (cationic), hydrophobicity, and amphipathicity. Structurally, they fall into two broad groups: linear *α*-helical and cysteine-containing forms with one or more disulfide bridges and *β*-hairpin-like, *β*-sheet, or mixed *α*-helical/*β*-sheet structures. The peptides assume these conformations upon contact with the target membranes [7–9]. Their characteristic physicochemical properties facilitate interactions with the phospholipid bilayer in the cell membranes of pathogens [10–12]. AMPs have been shown to kill pathogens directly by disrupting their membranes using mechanisms that are not fully understood. Several models, however, have been proposed. First, there is the “barrel-stave” model whereby a transmembrane pore is created by amphipathic *α*-helical peptides, disrupting the cell membrane of a pathogen. Second, the “carpet” model proposes that the peptides solubilize the membrane by interacting with the lipid head groups on the pathogen cell surface. This model was also proposed for viral killing [13]. Another is the aggregation model that is exhibited by sapecin from *Sarcophaga peregrina*, based on the existence of hydrophobic and hydrophilic domains on the AMPs. These structural features allow the peptides to form pores with hydrophilic walls and hydrophobic regions facing the acyl side chains of pathogen membrane phospholipids, thus facilitating movement of hydrophilic molecules through the pore [14]. Finally, the toroidal model, a subtle variation of the aggregation model, involves the formation of a dynamic lipid-layer core by hydrophilic regions of the peptide and lipid head groups and is induced by magainins, melittin, and protegrins [15–17]. While the indispensability of the structural features of cationic peptides in pathogen killing is under debate, charge differences between cationic peptides and lipids on the membrane are considered crucial. This may be the basis for their selective activity as nonhemolytic peptides have a high net positive charge distributed along the peptide length, whereas hemolytic peptides have a low negative charge [10, 11]. Evidence suggests that AMPs have intracellular targets. This is exemplified by elafin, a cationic and *α*-helical human innate defense AMP that does not lyse the bacterial membrane and is translocated into the cytoplasm. *In vitro* analysis using a mobility shift assay revealed that elafin binds DNA [18]. The histone-derived peptide buforin II binds nucleic acids in gel retardation assays and rapidly kills *Escherichia coli* by translocating into the cytoplasm of the pathogen and probably interfering with the functions of DNA or RNA. The structurally similar magainin 2 also kills *E. coli* but does not enter the cytoplasm [19]. Similarly, cationic antibacterial peptides enter the cytoplasm of *Aspergillus nidulans* and kill the fungus by targeting intracellular molecules whose identity has not been verified [20]. An excellent review of the intracellular targets of AMPs was recently published [21]. More studies are required, however, to confirm the existence and actual mode of action of AMPs with intracellular targets.

Insects produce AMPs constitutively at local sites or the AMPs are released systemically upon pathogenic infection to initiate pathogen-killing activities. In addition to the well-characterized *Drosophila* and mouse innate immune signaling pathways, the sequencing of the *Tribolium* genome has boosted research progress because bioinformatics analyses revealed putative immune-related genes based on comparisons with the genomes of other species [22].

AMPs are multifunctional molecules that, in addition to their well-known role as effectors of the innate immune system, are involved in several biologic processes and pathologic conditions, such as immune modulation, angiogenesis, and cytokine and histamine release [23–27]. Probably due to the negative charge in the plasma membrane of many cancer cells, some cationic peptides also have anticancer activity [28, 29]. These properties can be potentially exploited for clinical purposes [12, 30]. Cecropins are selectively cytotoxic to cancer cells, preventing their proliferation in bladder cancer, and are therefore likely candidates in strategies for the development of anticancer drugs [31]. In addition to antimicrobial activity, defensins facilitate the induction of adaptive immunity and promote cell proliferation and wound healing. Defensins show chemotactic activity whereby dendritic cells, monocytes, and T cells are recruited to the site of infection. Moreover, human *β*-defensins and the cathelicidin LL-37 stimulate the production of pruritogenic cytokines, such as interleukin-31, leukotrienes, prostaglandin E2, and others, suggesting an important role in allergic reactions [32–34]. AMPs also form the basis of the potentially lucrative commercial area of “cosmeceuticals”-products with beneficial topical activities that are delivered by rubbing, sprinkling, spraying, and so forth [35].

Here, we review the progress made in discovery of coleopteran AMPs, the molecular basis of *Tribolium* innate immunity, and prospects for the application of antimicrobial peptides in medicine.

## 2. The Discovery Process

### 2.1. Antimicrobial Peptides in *Tribolium*

The first wide-scale study of *Tribolium* immunity was conducted by Zou et al. in 2007 [22]. Taking advantage of the fully sequenced *Tribolium* genome to predict putative immune genes using bioinformatics techniques and real-time polymerase chain reaction (PCR), Zou et al. [22] predicted 12 AMPs in *Tribolium* compared to 20 in *Drosophila*, the most studied invertebrate. Another study using suppression subtractive hybridization led to the addition of a few more AMPs to this list [36] (see Table 1). Both studies identified four defensins in *Tribolium*, and phylogenetic analysis indicated that three of these are found in the evolutionary branch comprising only coleopterans. The fourth defensin (Def4) is found in a mixed branch that includes hymenopterans. A search of the Defensins Knowledgebase [37] revealed that the sequence information of this defensin is not available in the public domain, although its existence has been reported [22]. Attacins, which were identified in lepidopterans, were found in a cluster of three genes. Attacins are rich in glycine and proline, are structurally similar to coleoptericins, and are inducible by bacteria. Furthermore, *Drosophila* studies demonstrated that the induction of attacin is reduced in both *imd* and *Tl^−^* mutants [38]. Coleoptericins were first isolated from the larvae of *Allomyrina dichotoma* beetles immunized with *E. coli*. Coleoptericins also show activity against *Staphylococcus aureus*, methicillin-resistant *S. aureus*, and *Bacillus subtilis. *Like attacins, but unlike cecropins, coleoptericins do not form pores on the bacterial membrane, but do cause defects in cell division, as liposomes containing *E. coli* or *S. aureus* membrane constituents do not leak upon treatment with the recombinant form of coleoptericin, but instead form chains [39].

*Tribolium* cecropins are predicted to be pseudogenes because of a shift in the open reading frame; some cecropin-related proteins with an unusual structure, however, have been reported [22]. A cecropin has been reported in at least one coleopteran, *Acalolepta luxuriosa *[44].

Four thaumatin-like genes were found in *Tribolium* using suppression subtractive hybridization and genome search. Experimentally, septic injury induces thaumatin-1 and defensins in *Tribolium* [36]. Sterile wounding also induces thaumatin-1 and defensin-2. Furthermore, recombinant thaumatin-1 heterologously overexpressed in *E. coli* is active against fungi [36]. Coleopteran cationic peptides might be remarkably different from other known peptides and are therefore not readily identified by homology searches. A clear homolog of the *Drosophila* antifungal drosomycin could not be found in the *Tribolium* genome, but a weakly homologous protein with a cysteine-rich sequence was detected [22]. An overview of *Tribolium* AMPs indicates similarities with other coleopterans, but some differences with *Drosophila*. The work reported by these groups provides a good basis for advancing research on coleopteran AMPs.

### 2.2. Other Antimicrobial Peptides Identified in Coleopterans

A number of AMPs present in certain coleopterans have not yet been identified in *Tribolium* (see Table 2). One of these is an interesting class of insect peptides that adopts the knottin fold and was first identified in 2003 from the harlequin beetle, *Acrocinus longimanus*. Members of this class include Alo-1, Alo-2, and Alo3 [42]. Psacotheasin from the yellow star longhorn beetle *Psacothea hilaris* has also been identified as a member of this class [43, 45]. Alo-3 is active against fungi, while psacotheasin is active against bacteria and fungi. The knottin fold is characterized by a disulfide topology of the “*abcabc*” type, in which disulfide bridges are formed between the first cysteine and the fourth, second, and fifth cysteines, and the third and sixth cysteines [46]. Disulfide bridge formation may confer important properties to the peptides, such as stability and resistance to protease cleavage. Members of the knottin family in general have low sequence similarity, reducing their chances of identification by homology searches [46]. In contrast, however, the coleopteran knottin fold AMPs share sequence similarities with several plant antifungal peptides [42]. Although the mechanism by which these peptides function is not fully understood, psacotheasin kills *Candida albicans* by inducing apoptosis [47]. This has clinical significance as *C. albicans* can cause mild superficial to severe infections in immunocompromised patients. A better understanding of the molecular events that are critical to the induction of apoptosis by cationic peptides could lead to new targets for antifungal drug development. Alarmingly, candidemia, a systemic *Candida* infection, is on the increase and is accompanied by the reemergence of resistance against common drugs, pointing to the urgency of finding alternative means of treating fungal infections [48, 49].

### 2.3. Databases

The Antimicrobial Peptides database, a comprehensive and searchable database for AMPs was established based on information from literature surveys [50, 51]. Currently, an updated version on the website indicates that there are 1773 cationic peptides in the database, including antiviral (5.8%), antibacterial (78.56%), antifungal (31.19%), and antitumor (6.14%) peptides. Some of these peptides function against more than one type of pathogens. The structures of 231 of these peptides have been determined by nuclear magnetic resonance and X-ray diffraction studies. Another useful database is the Defensins Knowledgebase, which allows text-based searches for information on this large family of AMPs [37]. It is a manually curated and specialized database similar to the shrimp penaeidin database, PenBase [52]. We have also started molecular studies of another coleopteran, the dung beetle *Euoniticellus intermedius,* and sequenced the adult transcriptome with a view to study its immune system [53]. These databases serve as useful tools for the discovery and design of new peptides. Indeed, key features upon which antimicrobial activity is based have been studied using the Antimicrobial Peptides database [54, 55]. Such analyses generate an important information pool for drug design.

## 3. Regulation of AMP Expression by Coleopterans

**Figure 1:**
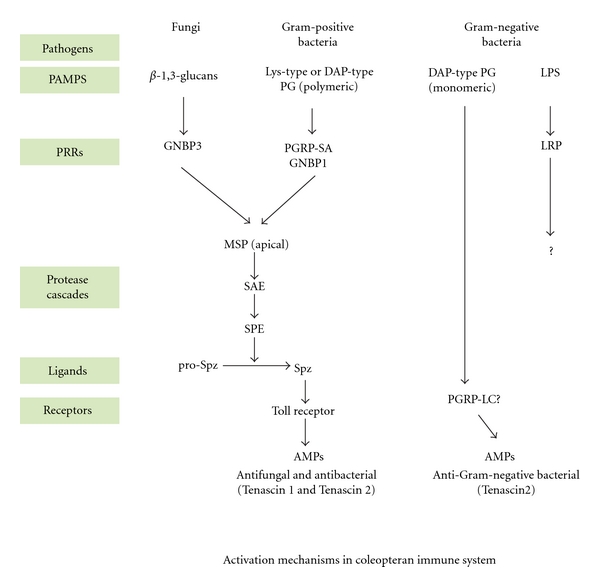
Activation mechanisms in the coleopteran immune system. Immune response pathways activated by bacteria and fungi showing a pathogen-associated recognition pattern (PAMP), pattern recognition receptors (PRRs), and downstream signaling molecules. The protease cascade in the Toll pathway involves the modular apical modular serine protease (MSP), the Spz-processing enzyme-activating enzyme (SAE), and the spaëtzle processing enzyme (SPE). GNBP3: glucan binding protein 3; PGRP: peptidoglycan recognition protein.

The signaling pathways that mediate the immune response in *Tribolium castaneum* were initially predicted based on a combination of *in silico* studies and experimental work by Zou et al. [22] and more recently another study involving the burying beetle *Nicrophorus vespilloides* [56]. In addition, studies using adult beetles exposed to *E. coli*, *M. luteus*, *C. albicans,* and *S. cerevisiae *have provided information on the signaling pathways. Accordingly, large-scale studies using real-time PCR revealed the presence of innate immune genes, such as PGRP-LA, PGRP-LE, PGRP-SA, PGRP-SB, several Toll proteins, and the immune deficiency (IMD) protein. Notably, some of the PGRPs had no orthologs in *Drosophila,* indicating a diversity of specificity. Recent biochemical studies using the large beetles *Tenebrio molitor* and *Holotrichia diomphalia *further elucidated the extracellular signaling network involved in responses to fungal and bacterial infections [41, 57]. Overall, coleopteran signaling appears to occur via the Toll and IMD pathways (Figure 1).

The Toll pathway is activated by PAMPS such as *β*-1,3-glucans, found in fungi, and by Lys-type peptidoglycans (PGN), found primarily in Gram-positive bacteria. A complex of the PAMPS and pathogen recognition receptors (PRRs) activates an apical protease, leading to a three-step serine protease cascade that culminates in the generation of active spaëtzle, the ligand of the transmembrane receptor Toll. Subsequent intracellular signaling leads to the transcriptional activation of genes that encode antimicrobial peptides.

Activation of the immune response by DAP-type PGN found primarily in Gram-negative bacteria and Gram-positive bacilli is still poorly understood in flies and beetles. Generally, it is understood that Gram-negative bacteria require the IMD pathway because *imd^−^* mutants cannot express antimicrobial peptides against Gram-negative bacteria. In *Drosophila,* candidates for the signal transduction-activated Gram-negative bacteria are the transmembrane receptor PGRP-LC and PGRPP-LE. Both molecules can activate the IMD pathway [3, 58]. Because these molecules are present in beetles and PGRP-LE is orthologous to the *Drosophila* protein, it is likely that the corresponding pathways are conserved. In *Tribolium*, PGRP-LA and PGRP-LE are activated by bacterial infection, but poorly activated by *C. albicans* and *M. luteus *[22]. Other *Tribolium* studies show that the IMD pathway is activated by two Gram-negative bacteria, *Xenorhabdus nematophila *and *E. coli, *inducing 12 AMPs of which 5 are significantly dependent on the IMD pathway as demonstrated by RNA interference studies [59]. The same study, however, demonstrated that two Gram-positive bacteria with different peptidoglycans expressed the same AMPs with only defensin-1 being dependent on Toll. Taken together, these studies show that while the pathways may be conserved, differences in PAMPS recognition and signal transduction exist between *Tribolium* and *Drosophila*.

The discovery of another PRR known as the LPS recognition protein (LRP) based on its *E. coli* agglutinating properties suggests the existence of an LPS pathway. LRP circulates in the hemolymph and does not agglutinate *S. aureus* or *C. albicans*. Interestingly, LRP comprises six repeats of an epidermal-growth-factor- (EGF)-like domain, an unusual structural feature for PRRs [60]. The downstream events in this pathway remain unclear.

## 4. Antimicrobial Peptides in Clinical Medicine

Cationic peptides have emerged as important targets for the development of therapeutics against bacteria, fungi, viruses, and parasites. They are key effector molecules in host defense through direct and indirect antimicrobial activity. Furthermore, in vertebrates, these peptides mediate a variety of cellular processes such as immunomodulation, wound healing, and tumorigenesis. These roles provide opportunities for the development of therapeutic products and vaccines. AMPs are attractive molecules for the development of clinical and veterinary therapeutics because they are fast acting and effective against susceptible pathogens, are less likely to cause the emergence of resistance compared to traditional antibiotics, have low toxicity to mammalian cells, and their mode of action tends to be more physical rather than targeted at metabolic pathways. A search of the FreePatentsOnline database using the word “antimicrobial peptide” produced more than 66.000 hits, and a number of AMPs have undergone clinical development [30]. A recent review of cationic peptides lists the peptides that are in various stages of clinical trials [29].

As mentioned above, the predicted *Tribolium* AMPs include defensins, attacin, coleoptericin, thaumatin, and cecropin. Defensins exhibit a broad spectrum of antimicrobial activity directed at bacteria, fungi, and viruses and are probably the most studied class of AMPs. Many therapeutic products have been modeled on them. The different types of defensins are either expressed constitutively or induced by infections to control the composition of microorganisms on surfaces such as the small and large intestines [61].

Many challenges remain that hamper the development of commercially viable peptides. The pressing issues concern pharmacokinetics (how the body deals with peptide drugs). When peptides are administered orally, the gastrointestinal tract may prevent their reabsorption into the systemic circulation. Furthermore, peptides may elicit an antigenic response when injected directly in the blood. This leaves topical medication the most feasible formulation while more research is being pursued to address the remaining obstacles. Despite these obstacles, the prospects for AMPs are not bleak because some have proceeded to clinical application. There is some optimism that these obstacles may soon be overcome by new strategies that combine natural cationic peptides and stable synthetic immunomodulatory peptides [29]. In this regard, peptide drugs such as Polymyxin B and gramicidin that are used for the treatment of Gram-negative bacterial infections are reported to be safe and effective, and peptides such as the indolicidin-derived CLS001 (previously known as MX594AN) have reached phase III clinical trials with promising prospects [62–64]. Because of their evolutionary distance, during which their survival against microbes has been solely dependent on innate immunity, insects provide interesting models for novel AMP drug design [65, 66].

## 5. Conclusions

The emergence of multidrug-resistant pathogens threatens human health globally and presents an urgent need to find antimicrobials with a reduced chance of inducing resistance. Cationic peptides for which the mechanism of action involves targeting the plasma membrane in a nonspecific manner, but does not involve specific proteins, offer good prospects. Admittedly, more work is needed to elucidate the mechanism of action of these peptides, as there is some evidence for intracellular targets. The importance of cationic peptides is further highlighted by their emerging prospects in other aspects of medicine, such as cancer treatment and vaccine development. Coleopterans are the most evolutionarily successful group of insects and are more representative of insects than *Drosophila*. In addition, human genes are more comparable to those of *Tribolium* than those of *Drosophila*. Thus, coleopterans are emerging as an important species for study as, like vertebrates, they have retained ancestral genes that are not present in *Drosophila*. Indeed, there is overwhelming evidence that coleopterans are more suitable for comparative studies between phyla than the commonly used dipterans. Here, we suggest that perhaps the outstanding evolutionary success of coleopterans is consistent with a robust immune system that warrants more attention than it has received to date.

## Figures and Tables

**Table 1:** Antimicrobial peptides currently predicted or identified in *Tribolium*.

Antimicrobial peptide	Accession number	Reference	Target	Method of identification
Attacin1	GLEAN_07737	[22]		Homology searches
Attacin2	GLEAN_07738	[22]		Homology searches
Attacin3	GLEAN_07739	[22]		Homology searches
Cecropin1	GLEAN_00499	[22, 31]	Antibacterial, antitumor	Homology searches
Cecropin2	Cec2	[22, 31]	Antibacterial, antitumor	Homology searches
Cecropin3	GLEAN_00500	[22, 31]	Antibacterial, antitumor	Homology searches
Defensin1	GLEAN_06250; XM_962101	[22, 36]	Antibacterial	Homology searches and suppression subtractive hybridization
Defensin2	GLEAN_10517; XM_963144	[22, 36]	Antibacterial	Homology searches and suppression subtractive hybridization
Defensin3	GLEAN_12469; XM_968482	[22, 36]	Antibacterial	Homology searches and suppression subtractive hybridization
Defensin4	Def4	[22]		Homology searches
Coleoptericin1	GLEAN_05093	[22]	Antibacterial	Homology searches
Coleoptericin2	GLEAN_05096	[22]	Antibacterial	Homology searches
Similar to thaumatin family	XM_963631	[36]	Antifungal	Suppression subtractive hybridization
Probable antimicrobial peptide	Tc11324	[22]		Homology searches
Putative antimicrobial peptide	AM712902	[36]		Suppression subtractive hybridization

**Table 2:** Antimicrobial peptides expressed in other coleopterans not yet identified in *Tribolium. *

Antimicrobial peptide	Organism	Accession no.	Reference
Diptericin A	*S. zeamis, (G. morsitans)*	Q8WTD5	[40]
Acaloleptin A	*S. zeamis (A. luxuriosa)*	Q76K70	[40]
Sarcotoxin II-1	*S. zeamis, (S. peregrina)*	P24491	[40]
Tenecin-1	*S. zeamis, (T. molitor)*	Q27023	[40, 41]
Tenecin-2	*T. molitor*		[41]
Luxuriosin	*S. zeamis, (A. luxuriosa)*	Q60FC9	[40]
Alo-3 (knottin type)	*A. longimanus*	P83653	[42]
Psacotheasin (Knottin type)	*P. hilaris*		[43]
